# Use of a Motorlance to Deliver Emergency Medical Services; a Prospective Cross Sectional Study

**Published:** 2019-08-21

**Authors:** Korakot Apiratwarakul, Kamonwon Ienghong, Thapanawong Mitsungnern, Praew Kotruchin, Pariwat Phungoen, Vajarabhongsa Bhudhisawasdi

**Affiliations:** 1 Department of Emergency Medicine, Faculty of Medicine, Khon Kaen University, Khon Kaen, Thailand.; 2 Research group for emergency patients care and emergency medical services, Faculty of Medicine, Khon Kaen University, Khon Kaen, Thailand.

**Keywords:** Emergency medicine, emergency medical services, ambulances, emergency mobile units

## Abstract

**Introduction:**

Access time to patients with critical or emergent situations outside the hospital is a critical factor that affects both severity of injury and survival. This study aimed to compare the access time to the scene of an emergency situation between a traditional ambulance and motorlance.

**Methods:**

This prospective cross sectional study was conducted on all users of emergency call, Srinagarind Hospital, Thailand, from June to December 2018, who received a registration number from the command center.

**Results:**

504 emergency-service operations were examined over a six-month period, 252 (50%) of which were carried out by motorlance. The mean activation time for motorlance and ambulance were 0.57 ± 0.22 minutes and 1.11 ± 0.18 minutes, respectively (p<0.001). Mean response time for motorlance was significantly lower (5.57 ± 1.21 versus 7.29 ± 1.32 minutes; p < 0.001). The response times during 6 a.m. to 6 p.m. were 5.26 ± 1.11 minutes for motorlance and 7.15 ± 1.39 minutes for ambulance (p < 0.001). These measures for night time (6 p.m. to 6 a.m.) were 5.58 ± 1.21 minutes and 8.01 ± 1.30 minutes, respectively (p < 0.001). The mean automated external defibrillator (AED) waiting time for motorlance and ambulance were 5.26 ± 2.36 minutes and 9.24 ± 3.30 minutes, respectively (p = 0.012). The survival rate of patients after AED use in motorlance and ambulance was 80% versus 37.5%; p<0.001.

**Conclusion:**

Emergency service delivery by motorlance had lower mean activation time, response time, AED time, and mortality rate of cardiac arrest patients compared to ambulance. It seems that motorlance could be considered as an effective and applicable device in emergency medical service delivery, especially in crowded cities with heavy traffic.

## Introduction

The access time of emergency medical services (EMS), to people who are injured or suffer from cardiac arrest is a critical factor that affects both severity of injury and survival (1-3). The use of traditional ambulances to reach people in emergency situations in large cities with traffic problems can result in delayed access times (4, 5). At present, there are efforts to choose vehicles that are flexible and can reduce access times such as motorcycles equipped with medical devices (motorlance) (6-8). Advanced emergency medical technicians (AEMTs) are responsible for driving the vehicle, which is able to measure oxygen saturation, check vital signs, and perform basic airway management and cardiopulmonary resuscitation (CPR). The driver must pass a motorcycle driving test administered at a standard training center and must use proper safety equipment at all times. In order to synchronize information with the command center, a headset-based radio is used, and the helmet microphone acts as a communication device. A motorlance can be used to send a doctor or nurse to the scene of severe traffic accidents quickly to administer treatment and deliver medical supplies such as blood or drugs (9). A previous study found that using a motorlance can reduce access times to injured persons in both urban and rural areas (10-13). However, there have been no studies about motorlance use in Thailand. The objective of the present study was to compare the access time to the scene of an emergency situation between a traditional ambulance and motorlance.

## Methods

***Study design and setting***

In this prospective cross sectional study, all users of emergency call (via emergency telephone number 1669 in Thailand operation), at Srinagarind Hospital, Thailand, from June 2018 to December 2018, who receive a registration number from the command center were evaluated. The present study protocol was approved by Khon Kaen University Ethics Committee for Human Research (HE611219). Requirement for informed consent from the patients was waived since patient confidentiality protection had been guaranteed, as patients were not identified by name, but by a unique study number.

***Participants***

Data of all patients aged over 18 years were collected. Cases in which the patient was being referred between hospitals and patients with missing data were excluded from this study.

***Data gathering***

Data were recorded using a standard national operation checklist for emergency medical services in Thailand consisting of demographic data (age, gender), operation time (day, night), type of patients (trauma, non-trauma), type of first procedure on scene for all patients, time stayed in emergency room, duration of hospital stay and outcome of patients with cardiac arrest who were referred by ambulance or motorlance.

A trained emergency medicine resident was responsible for data gathering under direct supervision of an emergency medicine specialist.

**Table 1:** Characteristics of the subjects and services

**Variable**	**Motorlance ** **(** **n=252** **)**	**Ambulance (n=252)**	**P value**
**Age ** **(** **years** **)**			
Mean ± SD	40.29±12.75	39.25±11.12	0.720
Median (25^th^, 75^th^ percentile)	40 (28, 50)	39 (27, 48)	
**Gender**			
Male	123 (48.8)	147(58,3)	0.548
Female	129 (51.2)	105 (41.7)	
**Operation time (shift)**			
8 a.m. to 4 p.m.	84 (33.3)	80 (31.7)	0.326
4 p.m. to 0.00 a.m.	131 (52.0)	135 (53.6)	
0.00 a.m. to 8.00 a.m.	37 (14.7)	37 (14.7)	
**Type of patients**			
Non-trauma	109 (43.2)	112 (44.4)	0.662
Trauma	143 (56.8)	140 (55.6)	
**First procedure on scene**			
Airway management	60 (23.8)	63 (25.0)	0.675
Breathing	104 (41.3)	110 (43.6)	0.359
Circulation	31 (12.3)	37 (14.7)	0.211
Immobilization	57 (22.6)	42 (16.7)	0.042*

Data are presented as mean ± standard deviation (SD) or number (%).

**Table 2:** Comparing the outcome between emergency services carried out by motorlance and ambulance

**Variable**	**Motorlance ** **(** **n=252** **)**	**Ambulance (n=252)**	**P value**
**Activation time (minutes)**			
Mean ± SD	0.57 ± 0.22	1.11 ± 0.18	p<0.001
**Response time (minutes)**			
Mean ± SD	5.57 ± 1.21	7.29 ± 1.32	< 0.001
6 a.m. to 6 p.m.	5.26 ± 1.11	7.15 ± 1.39	< 0.001
6 p.m. to 6 a.m.	5.58 ± 1.21	8.01 ± 1.30	< 0.001
**Duration of ED stay**			
< 1 hour	42 (16.7)	38 (15.1)	0.677
> 1 hour	210 (83.3)	214 (84.9)	0.680
**Duration of hospital stay**			
1 day	12 (4.8)	15 (6.0)	0.450
2 days	25 (9.9)	22 (8.7)	0.570
3 days	30 (11.9)	28 (11.1)	0.680
> 3 days	185 (73.4)	187 (74.2)	0.710
**AED waiting time** ^*^ ** (minutes)**			
Mean ± SD	5.26±2.36	9.24±3.30	0.012
**Mortality** ^*^			
Survived	8 (80.0)	9 (37.5)	<0.001
Not survived	2 (20.0)	15 (62.5)	

* For patients with cardiac arrest. AED: automated external defibrillator; ED: emergency department. Times from dispatch to resources being en route was defined as activation time and response time was defined as time from 1669 center call receipt to arrival on scene. Times from dispatch to AED arrival on scene defined as AED waiting time.

***Definitions***

Times from dispatch to resources being en route was defined as activation time and response time was defined as time from 1669 center call receipt to arrival on scene. Time from dispatch to AED arrival on scene was defined as AED waiting time.

***Statistical Analysis***

The sample size was calculated based on the standard deviation of access time detailed in a previous study by Peyravi (13). In order to achieve a significance level of 5% and power of test of 0.8, we determined that a sample size of 504 would be required. Statistical analysis was performed using SPSS for Windows version 16.0 (SPSS Inc., Chicago, IL, USA). Categorical data were presented as percentage, and continuous data were presented using mean and standard deviation. Univariate analysis was performed using two-sample t-test for numerical data and Chi-squared test for comparing data between the groups.

## Results

***Baseline characteristics of the subjects and services***

Five hundred four emergency-service operations were examined over a six-month period, 252 (50%) of which were carried out by motorlance. The characteristics of the subjects and services are shown in Table 1. The most common time of day at which motorlance was deployed was during the afternoon shift (4 p.m. to 0.00 a.m.; 52.0%). The most common first procedure performed on scene was breathing management. The procedure was performed in 41.3% of cases in the motorlance group compared with 43.6% in the ambulance group (p=0.359). 

***Outcomes***

Table 2 compares the outcomes between emergency services carried out by motorlance and ambulance. The mean activation time, mean response time (p<0.001), and mean automated external defibrillator (AED) waiting time (p = 0.012) were significantly lower in patients who were referred to ED by motorlance. The survival rate of cardiac arrest cases was significantly higher in motorlance group (80% versus 37.5%; p<0.001). There as not any difference between groups regarding duration of ED (p > 0.05) and hospital stays (p > 0.05).

## Discussion

Based on the findings of the present study, Emergency service delivery by motorlance had lower mean activation time, response time, AED time, and mortality rate of cardiac arrest patients compared to ambulance. 

Thailand's Emergency Medical Services (EMS) was established to allow equipment and medical staff to be deployed to manage and treat emergency patients en route using a traditional ambulance. Emergency medical responders (EMRs), emergency medical technicians (EMTs), advanced emergency medical technicians (AEMTs), and paramedics are deployed through this service. Physicians are in short supply and mainly provide services in the emergency room and command center with online medical oversight (11). Thailand will enter the 4.0 era in accordance with government policy. Public health development is a branch that must develop for taking care of people with international standards. Caring for patients who are injured at the scene through the operation of emergency medical services is important to reduce the mortality rate (14).

Response and activation times are key factors in accessing emergency patients. In major cities, traffic congestion can negatively affect the arrival time of emergency services to the scene of an emergency. Motorlance can, thus, be used to reduce arrival times and increase the efficiency of Emergency Medical Services.

Although there are several medical emergency systems around the world that use motorlance, few studies have been published that evaluate their effectiveness (1). The main objective of using motorlance is to reduce the time it takes to administer treatment in critical situations. Our study, which examined 504 cases over a six-month period, found that the average motorlance response time was shorter than that of traditional ambulances (5.57 versus 7.29 minutes), a result that is consistent with those of previous studies conducted in various countries (5-8).

Response time and use for standard emergency medical services is related to the infrastructure of the city and the availability of different hospitals combined with low traffic congestion (10). In Thailand, the maximum response time in resuscitation cases is set at eight minutes. The use of a motorlance allowed us to achieve this goal. In addition, emergency medical services need to reach patients quickly to assess symptoms and provide treatment. Medical staff will operate by using an ambulance as a travel vehicle, the signal lights and the siren sound of ambulance cause drivers to make way for ambulance (15).

After delivery to hospital, the time that patients stayed in emergency room (ER), which depends on many steps of work (time to doctor visit, overcrowding in ER, blood test waiting time), was not different between motorlance and traditional ambulance; a result that is consistent with those of previous studies (16), which showed patient disposition, especially admission, investigation and triage level were the main factors leading to longer periods in emergency department. Of course, the duration of hospital stay also depends on multiple factors including severity of disease.

In cardiac arrest patients, AED waiting time was shorter in motorlance group (5.26 versus 9.24 minutes), resulting in a higher survival rate among patients after AED use in motorlance compared to ambulance (80% versus 37.5%). Basic life support (BLS) and AED usage is one of the first aid procedures that general population can legally perform in Thailand. Teaching these procedures to the general population will increase survival rate of cardiac arrest patients (17).

As a result of this study, the National Institute for Emergency Medicine (NIEM), which is responsible for administrative management and coordination between relevant agencies in both public and private sectors, will promote the use of motorlance for delivering emergency medical services in Thailand.

## Limitation:

The use of the motorlance was limited in some weather conditions (heavy rain). It should be noted that in the current study, data were gathered from only one emergency medical service center. 

## Conclusion:

Emergency service delivery by motorlance had lower mean activation time, response time, AED time, and mortality rate of cardiac arrest patients compared to ambulance. It seems that motorlance could be considered as an effective and applicable device in emergency medical service delivery, especially in crowded cities with heavy traffic.
